# Beetle larvae with unusually large terminal ends and a fossil that beats them all (Scraptiidae, Coleoptera)

**DOI:** 10.7717/peerj.7871

**Published:** 2019-10-14

**Authors:** Joachim T. Haug, Carolin Haug

**Affiliations:** 1 Department of Biology II, Ludwig-Maximilians-Universität München, Planegg-Martinsried, Germany; 2 GeoBio-Center at LMU, München, Germany

**Keywords:** Larval diversity, Baltic amber, False flower beetle, Combinatorial morphospace, Fossil larva

## Abstract

Larvae, and especially fossil larvae, are challenging to deal with from a purely taxonomic view. Often one cannot determine which species the larvae belong to. Yet, larvae can still contribute to various scientific questions. Especially morphological traits of a fossil larva can be highly informative for reconstructing character evolution. Also the occurrence of specific larval types and larval characters in time and the disappearance of such forms can well be reconstructed also without being able to narrow down the phylogenetic relationship of a larva very far. Here, we report two new beetle larvae preserved in Baltic amber which are identified as representatives of Scraptiidae, based on an enlarged terminal end (‘9th abdomen segment’); this is only the third record of such larvae. In comparison to modern forms, the terminal ends of the two new fossil larvae is even larger in relation to the remaining body than in any known larva. Unfortunately, our knowledge of such larvae in the modern fauna is very limited. Still, one of the two already known fossil larvae of Scraptiidae also has a very long terminal end, but not as long as those of the two new fossils. These three fossil larvae therefore seem to possess a specific morphology not known from the modern fauna. This might either mean that they (1) represent a now extinct larval morphology, a phenomenon well known in other euarthropodan lineages, or that (2) these forms represent a part of the larval phase not known from modern day species as they have not been described yet; such cases occur in closely related lineages. In any case, the fossils expand the known diversity of larval morphologies.

## Introduction

Zoological research is in general heavily centred around adult individuals. There are, of course, exceptions to this, e.g. embryology or evo-devo, but for many sub-fields of zoology this is definitely the case (Minelli et al., 2006). This may be coupled to the fact that in many cases zoological thinking is focussing on taxonomic units, mostly species. Adults can much easier be identified to species level than non-adults as even in the age of DNA barcoding morphological characters are still used as the major tool for identifying specimens.

This situation is unfortunate as immatures, especially larvae (see Haug, 2018 for challenges of this term), represent an important part of the life time of an organism and often fulfil a different ecological role than the adult. As zoological research is adult centred we often lack such information on larvae. This does not only account for modern day metazoans, but also for fossil representatives.

Though morphology is still the prime method for identifying extant and fossil species, it can also be used for other aspects of an animal. It is possible to recognise a larva as something special, even without being able to narrow down its taxonomic identity very far. Especially for representatives of Insecta and their relatives there are numerous examples of larvae that cannot be easily taxonomically treated, but still provide important information for various zoological questions:

1. A larva may possess an unusual, so far unknown or unrecognised overall morphology (Williamson, 1960; Henry, 1978; Gamô, 1979; Martin & Ormsby, 1991; Chen et al., 2014; Haug & Haug, 2014; Haug et al., 2016a; Rudolf, Haug & Haug, 2016).
2. A larva may possess a combination of characters so far unknown or unrecognised for a specific larval stage (Villamar & Brusca, 1988; Lindley et al., 2002; Haug et al., 2016b).
3. Larvae may possess much more variability among each other than expected based on the variability of the adults (De Beer, 1958; Lang, Krapp & Melzer, 2007; Hübner et al., 2017).
4. Fossil larvae may provide minimum ages for specific larval types known from the modern fauna (Maisey & De Carvalho, 1995; Kadej & Háva, 2011; Waloszek & Dunlop, 2002; Briggs et al., 2005; Pohl, 2009; Wang & Zhang, 2010; Zhang et al., 2010; Makarkin, Wedmann & Weiterschan, 2012; Haug, Wiethase & Haug, 2015; Haug, Martin & Haug, 2015; Serrano-Sánchez et al., 2016; Néraudeau et al., 2017; Haug, Müller & Haug, 2018; Pérez-De La Fuente et al., 2018). This may well also represent a case of the oldest representative of a lineage, i.e. provide a taxonomic or phylogenetic signal as well.
5. Fossil larvae may possess more plesiomorphic characters no longer represented in the modern fauna, providing important clues for reconstructing character evolution (Müller & Walossek, 1986a, 1986b; Walossek & Müller, 1990; Wang, Ponomarenko & Zhang, 2009; Godunko, Staniczek & Bechly, 2011; Haug & Haug, 2013, 2015, 2016; Haug et al., 2013a, 2015; Badano et al., 2018).
6. Fossil larvae of such more ancestral forms (under point 5) may persist longer in time than at first expected (‘morpho-type survival’ in the sense of Haug et al., 2012) extending the range of such larval morphologies (Kukalova-Peck, 1978; Godunko, Staniczek & Bechly, 2011; Haug, Haug & Garwood, 2016).
7. Fossil larvae may possess characters today only known from different modern lineages, but not occurring together in the same specimen, i.e. represent a specific combination unknown today (Badano et al., 2018; Haug et al., 2019; Haug, Müller & Haug, 2019).
8. Larvae may possess unusually sized body parts. Unlike most of the cases above, which are easily recognisable based on qualitative character, this refers to quantitative differences (Haug, Müller & Haug, 2019, in review a, in review b).

Here, we report two unusual appearing beetle larvae preserved in Baltic amber, representing a case 8. The terminal end is unusually large compared to that in modern day forms. We use a quantitative approach to evaluate the exceptionality of this find.

## Materials and Methods

### Material

Two specimens preserved in amber were bought from an amber trader from Vilnius, Lithuania (ambertreasure4u.com). Amber pieces were already prepared to a high quality when bought. Specimens are now deposited in the Palaeo-Evo-Devo Research Group Collection of Arthropods, Ludwig-Maximillians-University of Munich, Germany, under repository numbers PED 0006 and PED 0011. According to the trader, both pieces are Baltic amber.

### Documentation methods

Both specimens were documented on a Keyence VHX-6000 digital microscope. Magnifications higher than 300× did not result in a higher resolution of details in the image due to optical properties of the amber. To overcome limitations in depth of field, image stacks were recorded with changing levels of focus. Stacks were fused with the built-in software. To overcome limitations of field of view, several adjacent image details were recorded, each with a stack of images (composite imaging; Haug, Haug & Ehrlich, 2008; Haug et al., 2011). Fused images were merged into panorama images with the built-in software. Each image was additionally recorded under different exposure times (HDR; Haug et al., 2013b; Haug, Müller & Haug, 2018). Specimens were documented from both sides, in front of black background and white background, with cross-polarised coaxial illumination and with unpolarised ring illumination. The images with most information were chosen for presentation in this publication.

### Measurements

Several dimensions were measured on the specimens as well as on all comparable specimens in the literature. Measured dimensions include total body length l(tot), length of head l(h), length of thorax l(th), combined length of head and thorax l(h+th), length of terminal end l(te), maximum width of head w(h), maximum width of trunk w(tr), maximum width of terminal end w(te). As not all specimens in the literature have been provided with a scale, we could not always use absolute values for comparison. Additionally, we calculated ratios; each measured dimension was divided by the total body length.

All ratios are shown as a parallel coordinate plot with the absolute ratios (combinatorial morphospace from Mander, 2016). Additionally, relative ratios were calculated by dividing each value for each ratio by the maximum value for each ratio. This provides a better spreading of the values. Also this plot is shown as a parallel coordinate plot. Additionally, biplots (scatter plots) are provided for some ratios.

We additionally calculated the absolute lengths where scales or lengths were available to get at least a rough impression about growth. Yet, some of these need to be seen as estimations as the values given in the literature were not always very exact. Therefore, the ratios are seen as the more reliable (and larger) data set. All values in Table S1.

### Terminology

Insecta is an ingroup of Crustacea s.l.; this seems largely undisputed. Among crustaceans there are many highly specialised terms within different ingroups. Still, there is a more general type of neutral terminology that can be applied to enhance communication across group boundaries. Consequently, this should also be true for Insecta. We therefore provide here terms specific for describing representatives of Insecta along with a more general crustacean-type terminology (these are provided in squared brackets). Description follows the principles lain out in Haug, Briggs & Haug (2012), yet provided here as plain text for convenience.

## Results

### Description of specimen PED 0006

**Figure 1:**
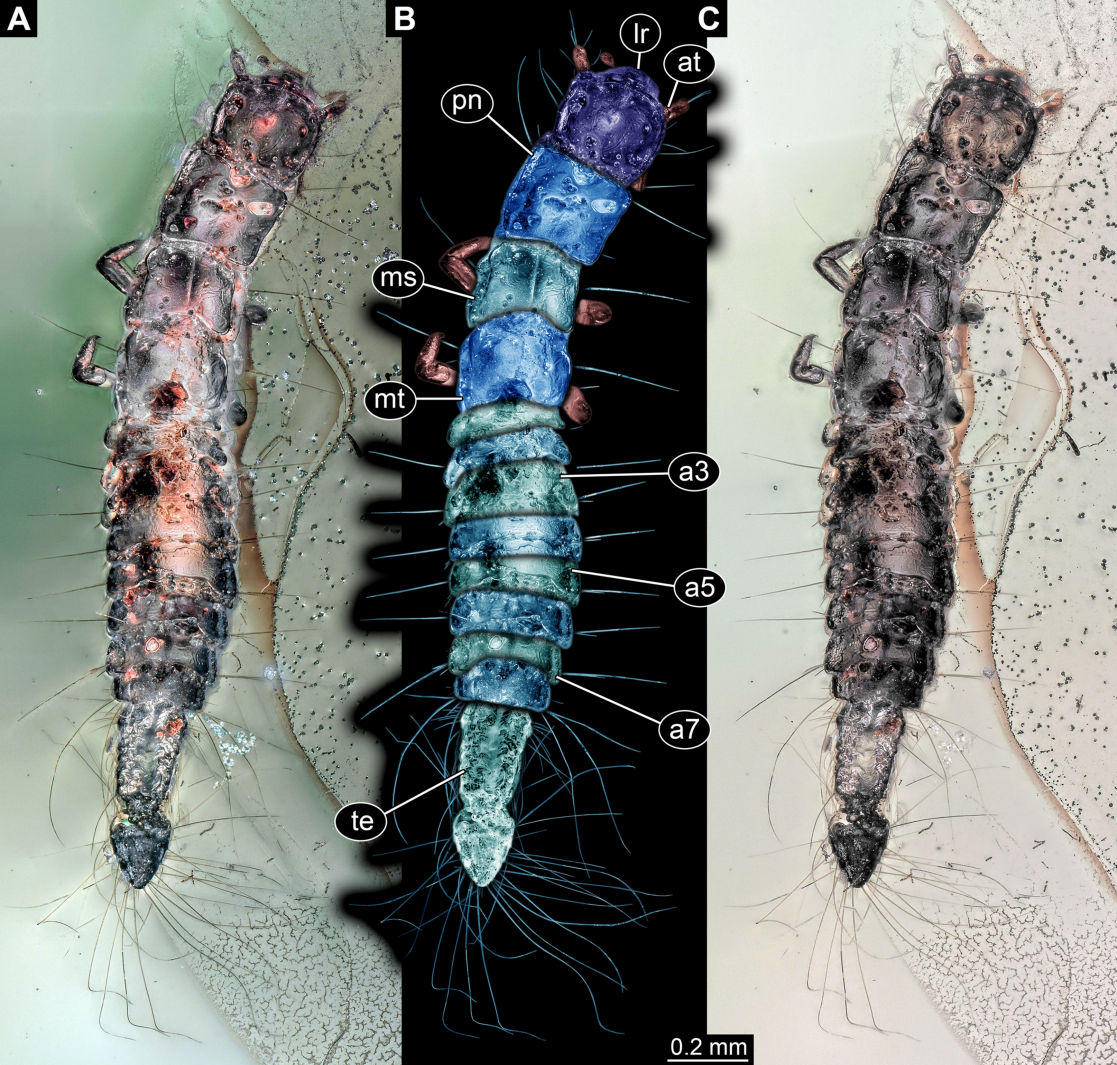
Larva of Scraptiidae preserved in Baltic Amber, specimen TripleB1 (PED 0006). All composite images in dorsal view. (A) Cross-polarised co-axial light. (B) Colour-marked version of (A). (C) Non-polarised ring illumination. Abbreviations: a3–7, abdomen segment 3–7; at, antennula; lr, labrum; ms, mesonotum; mt, metanotum; pn, pronotum; te, trunk end.

**Figure 2:**
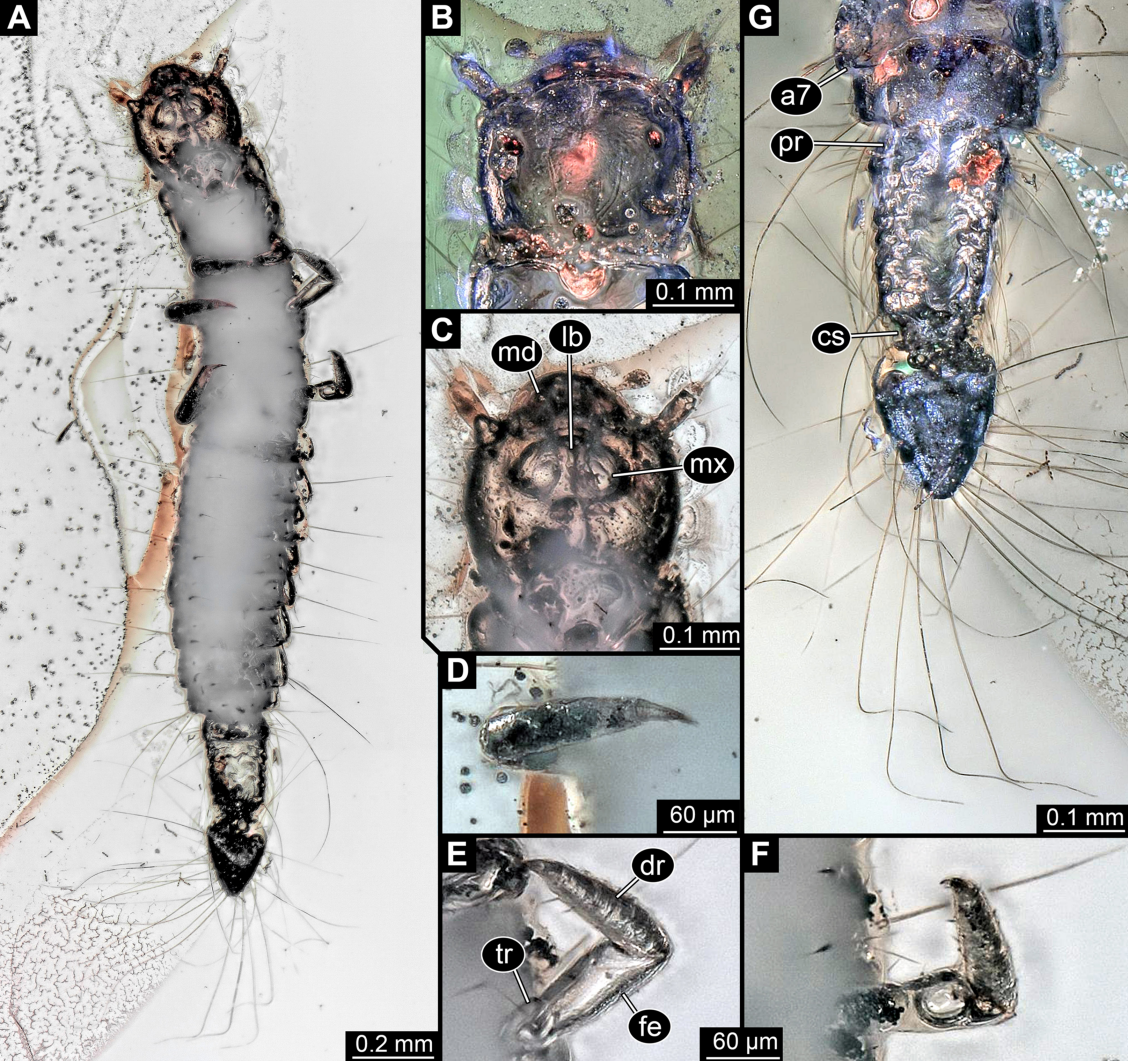
Larva of Scraptiidae preserved in Baltic Amber, specimen TripleB1 (PED 0006), continued. All composite images. (A) Ventral view, non-polarised ring illumination. (B–G) Close-ups. (B) Head in dorsal view, cross-polarised co-axial light. (C) and (D) Non-polarised ring illumination. (C) Head in ventral view. (D) Right appendage of mesothorax (“midleg”). (E) Left appendage of mesothorax (“midleg”). (F) Left appendage of metathorax (“hindleg”). (G) Terminal end in dorsal view. Abbreviations: a7, abdomen segment 7; cs, constriction; dr, distal region; fe, femur; lb, labium; md, mandible; mx, maxillula; pr, proximal region; tr, trochanter.

**General habitus:** Body elongate, organised into 13 discrete units (Fig. 1), overall length about 2.05 mm. First unit is the head, supposedly including six segments, ocular segment plus five post-ocular segments. Remaining units forming trunk. Anterior trunk or thorax with three distinct sub-similar units representing true segments. Remaining nine units presumably representing eight segments and prominent terminal end (Fig. 2A), together forming posterior trunk or abdomen (not corresponding to abdomen in other crustacean groups). Dorsal surface with numerous bubble-like structures (Fig. 2B), most likely representing artefacts, not true structures of the original morphology.

**Head:** Forming capsule, in dorsal view about as long as wide (Fig. 2B). With about three long setae along the lateral sides. V to U-shaped lines apparent dorso-posteriorly, representing moulting suture.

Ocular segment recognisable by anterior structure, labrum, or clypeo-labral complex [hypostome-labrum complex] (Fig. 1B). Post-ocular segment 1 recognisable by its appendages, antennae [antennulae]. Details difficult to access; stout bulbous, more than 2.5× as long as wide. Very proximal region partly set off indicating subdivision, yet unclear if already functional. At least three long setae distally, one additional one further proximally (Fig. 2B). Post-ocular segment 2, intercalary segment, not recognisable by outer structures.

Post-ocular segment 3 recognisable by its appendages, mandibles. Only faint outlines apparent dorsally concealed by labrum (Fig. 2C).

Post-ocular segment 4 recognisable by its appendages, maxillae [maxillulae]. More prominent than mandibles, bulging proximally, distal part only faintly visible (Fig. 2C).

Post-ocular segment 5 recognisable by its appendages, labium [maxillae]. Only faint outline apparent between the maxillae (Fig. 2C).

**Anterior trunk, thorax:** Post-ocular segment 6, thorax segment 1, prothorax, dorsally forming prominent sclerite, tergite, pronotum. Slightly shorter than head, about as wide as head. Ventral details largely obscured by Verlumung. Ventro-laterally with prominent pair of appendages (legs, front legs), one appendage on each side (Fig. 2A). Proximal parts largely obscured, but apparently organised into at least 4 discrete elements. Most proximal visible element (trochanter?) short, about as long as wide. Visible element 2, most likely the femur, widening distally, more than 2× as long as wide. With a prominent membranous ‘window’ disto-medially. Distal region of appendage as long as femur plus possible trochanter. Distally claw-like element set off from rest (‘tarsungulum’). Unclear whether corresponding to future tarsus or only to part of it. Hence unclear whether proximal part of distal region corresponding to tibia or tibia plus parts of tarsus.

Post-ocular segment 7, thorax segment 2, mesothorax, sub-similar to preceding segment in most aspects (Figs. 1, 2D and 2E). Tergite, mesonotum, slightly shorter than pronotum. Post-ocular segment 8, thorax segment 3, metathorax, sub-similar to preceding segment (Figs. 1 and 2F).

All three thorax segments with one prominent laterally projecting seta on each side (Fig. 1). Seta about as long as segment wide. Seta on prothorax very far anterior, on mesothorax and metathorax about in the middle along the anterior-posterior axis.

**Posterior trunk, abdomen:** Post-ocular segment 9, abdomen segment 1, dorsally with distinct tergite (Fig. 1). Shorter than that of preceding segment, less than 30%. Laterally slightly drawn out into small paranotal lobes (tergo-pleura). Slightly wider than preceding segment. Post-ocular segment 10, abdomen segment 2, sub-similar to preceding segment, slightly wider. Post-ocular segment 11, abdomen segment 3, sub-similar to preceding segment, slightly longer and wider. Post-ocular segments 12–16, abdomen segments 4–8, sub-similar, shorter than abdomen segment 3 but longer than 2; progressively decreasing in width along the series. Abdomen segments 1–7 with one prominent laterally projecting seta on each side. Seta about as long as segment wide. One or two smaller accompanying setae visible on most segments, inferred to be present in all. Abdomen segment 8 with three long setae and accompanying setae. Two of the three long setae prominently longer than the third one, at least 1.5× as long as the third one.

Trunk end articulating to abdomen segment 8. Long, about as long as abdomen segments 4–8 combined. Roughly differentiable into two regions (Fig. 2G). Anterior region about 60% of the length, almost rectangular in dorsal view, only slightly narrower posteriorly. Set off from posterior region by distinct constriction. Posterior region roughly heart-shaped (Fig. 2G).

In the far anterior region of the trunk end with about seven rather short setae along the lateral edge on each side. Seta maximally as long as length of a single abdomen segment. In the further posterior region of the anterior region with about nine longer setae. Setae about 2× as long as those of the very anterior part. The heart-shaped posterior region bears about 16 very long setae along each lateral side. Setae longer than any other seta, about 2× as long as the long seta on the abdomen segments.

### Description of specimen PED 0011

**Figure 3:**
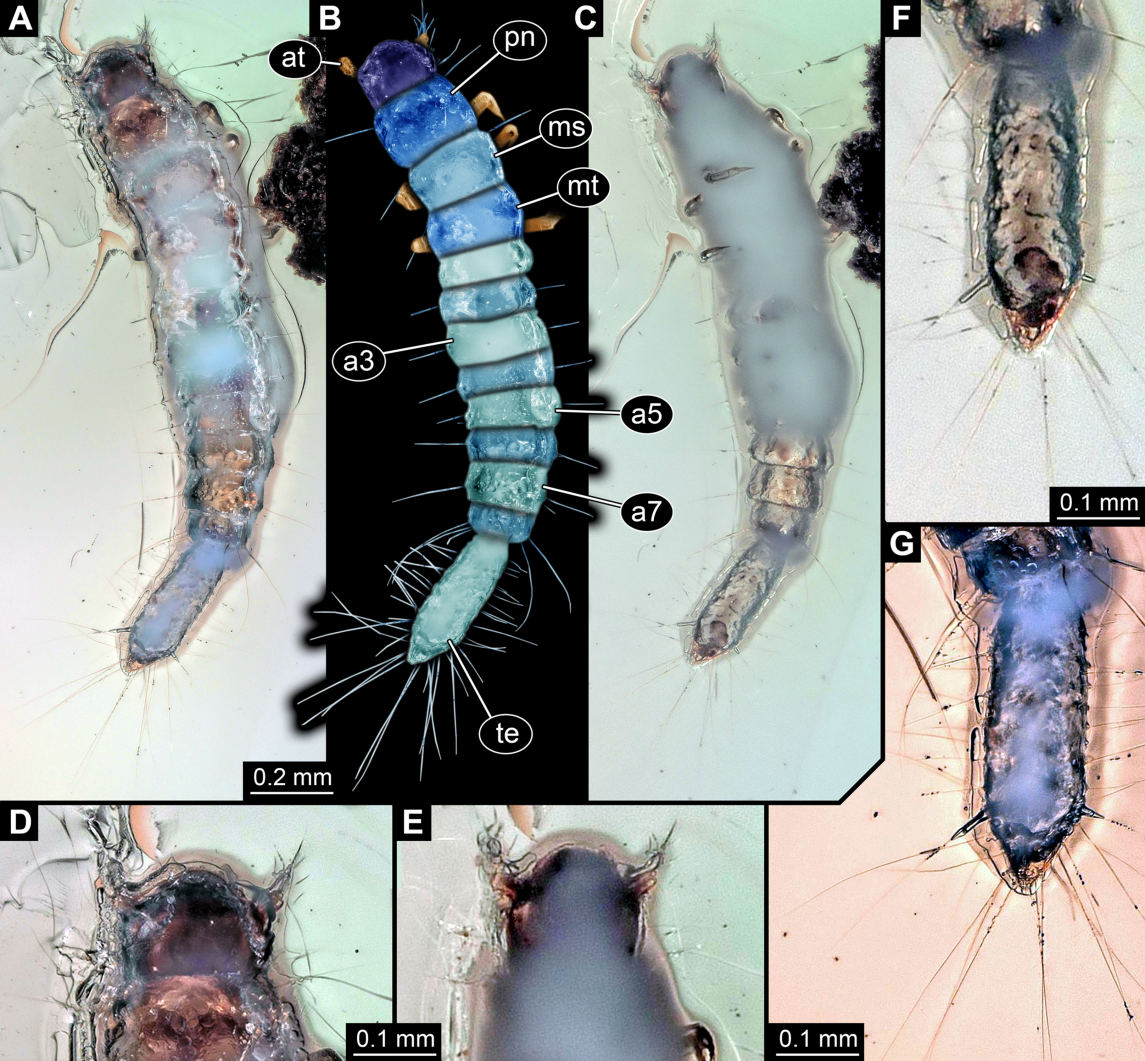
Larva of Scraptiidae preserved in Baltic Amber, specimen TripleB2 (PED 0011). All composite images. (A–F) Cross-polarised co-axial light. (A) Dorsal view. (B) Colour-marked version of (A). (C) Ventral view, image flipped. (D–G) Close-ups. (D) and (E) Head. (D) Dorsal view. (E) Ventral view. (F) and (G) Terminal end. (F) Ventral view, image flipped. (G) Dorsal view, non-polarised ring illumination. Abbreviations: a3–7, abdomen segment 3–7; at, antennula; ms, mesonotum; mt, metanotum; pn, pronotum; te, trunk end.

**General habitus:** Body elongate, organised into 13 discrete units, overall length about 1.49 mm (Figs. 3A and 3B). First unit is the head, supposedly including six segments, ocular segment plus five post-ocular segments. Remaining units forming trunk. Anterior trunk or thorax with three distinct sub-similar units representing true segments. Remaining nine units presumably representing eight segments and prominent terminal end, together forming posterior trunk or abdomen (not corresponding to abdomen in other crustacean groups). Dorsal surface partly concealed by Verlumung, ventral side even more so (Fig. 3C).

**Head:** Forming capsule, in dorsal view wider than long, less than 1.5× (Fig. 3D). No further details available of the head capsule.

Ocular segment indicated by anterior structure, labrum, or clypeo-labral complex [hypostome-labrum complex]. Post-ocular segment 1 recognisable by its appendages, antennae [antennulae]. Details difficult to access; stout bulbous. Very proximal region partly set off indicating subdivision, yet unclear if already functional. Setae present, details not accessible (Figs. 3D and 3E). Post-ocular segment 2, intercalary segment, not recognisable by outer structures. Details of post-ocular segments 3–5, i.e. their appendages not accessible as concealed by Verlumung (Fig. 3E).

**Anterior trunk, thorax:** Post-ocular segment 6, thorax segment 1, prothorax, dorsally forming prominent sclerite, tergite, pronotum. Slightly longer than head, also wider than head. Ventral details largely obscured by Verlumung. Ventro-laterally with prominent pair of appendages (legs, front legs), one appendage on each side. Proximal part largely obscured, but apparently organised into at least three discrete elements (Fig. 3C). Most proximal visible element, femur, only partly visible. Distal region of appendage subdivided. Distally claw-like element set off from rest (‘tarsungulum’). Unclear whether corresponding to future tarsus or only to part of it. Hence unclear whether proximal part of distal region corresponding to tibia or tibia plus parts of tarsus.

Post-ocular segment 7, thorax segment 2, mesothorax, sub-similar to preceding segment in most aspects (Figs. 3A and 3B). Tergite, mesonotum, slightly shorter than pronotum. Post-ocular segment 8, thorax segment 3, metathorax, sub-similar to preceding segment. All three thorax segments with one prominent laterally projecting seta on each side. Seta shorter than segment wide. Setae far anterior on the segments.

**Posterior trunk, abdomen:** Post-ocular segment 9, abdomen segment 1, dorsally with distinct tergite. Shorter than that of preceding segment, about 50%. About as wide as preceding segment. Post-ocular segment 10, abdomen segment 2, sub-similar to preceding segment, slightly wider. Post-ocular segment 11, abdomen segment 3, sub-similar to preceding segment, slightly longer and wider. Post-ocular segments 12–16, abdomen segments 4–8, sub-similar, shorter than abdomen segment 3 but longer than 2; progressively decreasing in width along the series. Abdomen segments 1–7 with one prominent laterally projecting seta on each side. Seta about as long as segment wide. One or two smaller accompanying setae visible on most segments, inferred to be present in all. Abdomen segment 8 with three long setae and accompanying setae. Two of the three long setae prominently longer than the third one, at least 1.5× as long as the third one.

Trunk end articulating to abdomen segment 8. Long, slightly longer than abdomen segments 5–8 combined. Elongate pentagon-shaped in dorsal view (Figs. 3F and 3G). Two vertices of the pentagon forming the anterior rim of the terminal end. Two further posterior vertices at about 75% of the length (anterior-posterior axis). Lateral distance between the two vertices slightly larger than that between the two anterior vertices. Fifth vertex forming the posterior terminal apex. Numerous setae along the lateral margin. About eleven setae between one anterior vertex and further posterior vertex, increasing in size towards posterior. Most posterior ones of these about as long as the ones arising from the anterior abdomen segments. About ten setae between one further posterior vertex and the posterior terminal vertex. Setae longer than the further anterior ones, at least 1.5×.

## Discussion

### Systematic and taxonomic interpretation of the specimens: coarser frame

The overall morphology, i.e. the tagmosis with head, thorax of three segments and trunk without walking appendages (abdomen), of the two fossils immediately identifies them as representatives of Insecta. The lower number of abdomen segments, the softness of the segments, the short head structures and the structure of the thorax appendages indicate that the specimens are larvae of a holometabolan species.

The head morphology of the specimens is best compatible with an interpretation as beetle larvae. The extremely prominent terminal end of both specimens in combination with a rather worm-shaped body has been suggested to be characterising for the beetle group Scraptiidae (Klausnitzer, 1978).

### Biology and taxonomy of Scraptiidae

Scraptiidae, the group of false flower beetles, has been named by Mulsant (1856) and comprises currently about 400 species, organised into about 30 monophyletic groups that are generally accepted at genus level. Scraptiidae is an ingroup of Cucujiformia and furthermore of Tenebrionoidea. It is nowadays furthermore organised into two supposedly monophyletic groups, Scraptiinae and Anaspidinae (both names likewise introduced by Mulsant, 1856). Anaspidinae had formally been suggested to represent an ingroup of Mordellidae, but was re-interpreted as an ingroup of Scraptiidae (Crowson, 1953; Franciscolo, 1954). Also supposed representatives of other groups have over the years been recognised as representatives of Scraptiidae (Watt, 1987; see review in Young, 1987). Scraptiidae was not recovered as monophyletic in the large-scaled analysis by Lawrence et al. (2011), yet so far no further taxonomic consequences appear to have been drawn from this finding. Lawrence et al. (2011) also discussed possible causes of artefacts in their study. It therefore does not appear that the group is currently considered non-monophyletic and we treat Scraptiidae here for simplicity as possibly still being monophyletic.

The biology of many species of the group is basically unknown (Hangay & Zborowski, 2010). Adults, especially of the group Anaspidinae, appear to be often found in large number in different types of flowers (Young, 1987), hence the vernacular name false flower beetles. Some species appear to be associated with lichens (Buck, 1954; Hayashi, 1962).

Within Scraptiidae not all larvae have a bulbous terminal end similar to that of the fossils. The larvae of species of *Anaspis* have paired terminal ends (Watson & Dallwitz, 2003), unlike those in the fossils and those of extant larvae of the group *Scraptia*. Of most of the other ingroups of Scraptiidae the larvae seem unknown. A possible larva of a species of *Canifa* was reported on the website ‘bugguide’ (see further below for details). The terminal end of this larva in principle resembles those of the larvae of the group *Scraptia*. Also Watt (1987) states that the larvae of *Nothotelus* resemble those of *Scraptia*. One could therefore guess that the prominent terminal end is a feature characterising larvae of Scraptiinae, yet we do not seem to know the exact larval morphology of many species of Scraptiidae, therefore, this must remain partly unclear.

### The fossil record of Scraptiidae

Willemstein (1987) suggested that representatives of Scraptiidae should have been present in the Cretaceous. Representatives of supposedly closely related groups are present in deposits of this age (Peris & Ruzzier, 2013). In younger Baltic amber there have been reports of at least eight species, based on adults, of Scraptiidae (reviewed in Perkovsky & Odnosum, 2009; Alekseev, 2013). It appears that even more species are represented, but the material has not been intensely studied. More adult specimens have for example been reported by Kubisz (2000, 2001). Even two larvae in Baltic amber have already been described before (Weitschat & Wichard, 2002, their fig. 63E; Gröhn, 2015, his fig. 7691, p. 280).

In holometabolan representatives of Insecta the morphology of the larvae is strongly differentiated and decoupled from that of the adults. This makes describing new species based on larvae challenging at best. Yet, it is a common strategy. If a reliable differential diagnostic can be provided also larvae can represent stable and usable types. This is also true for fossils (compare e.g. Badano et al., 2018; Pérez-De La Fuente et al., 2018). In the current case at least four formally described species may represent the adult form of the here reported larvae. Without a pupa, ideally emerging from a larval exuvium, it is factually not possible to decide to which of the four species these larvae should be attributed. It is even not possible to provide a proper differential diagnosis comparing the larvae with extant ones, as it is well possible that these are not of corresponding stages. Therefore, possible differences may simply represent ontogenetic differences (see further below for details).

### Taxonomy of the new larvae

As pointed out above, the larvae cannot be narrowed down strictly within Scraptiidae, only the ingroup *Anaspis* can be reliably excluded. More precisely, the taxonomic distinction can be summarised as (following Schädel, Müller & Haug, in press):

Scraptiidae nec. *Anaspis*

No further reaching systematic of taxonomic interpretation is possible, as long as our knowledge on extant larvae is that incomplete. The two larvae will be referenced by a short nickname to allow a quick recognition without erecting a para-taxonomy (Haug et al., 2016a). We refer in the following to the specimens PED 0006 as TripleB larva 1 and to PED 0011 as TripleB larva 2, as short forms for ‘big butt beetle larva’.

### Biology of larvae of Scraptiidae

As for many other ingroups of Insecta also the larvae are only known for quite few representatives, and mostly for species of *Scraptia* (Böving & Craighead, 1931; Klausnitzer, 1978; Lawrence et al., 2011; see also further below). Unfortunately, many reports in the literature are just re-drawings from older sources and hence the true number of known larvae is lower than the references suggest (see below for details). Even more so, the biology of the larvae is only known very scarcely. Most larvae of the group Tenebrionoidea (of which Scraptiidae is an ingroup) are generally considered to be saproxylic (Majka & Pollock, 2006). Hence it is not surprising that larvae of Scraptiidae have been found in decaying wood (Vanin et al., 1996), making it likely that this applies for all these larvae. Saproxylic organisms are in fact abundant and common in different types of amber (Peris et al., 2016; Kraemer et al., 2015, 2018). Whether there is a specific function of the enlarged terminal end coupled to the saproxylic life style is not known; obviously, the larvae are able to autotomise it (Švácha, 1995).

### Extant larvae of Scraptiidae with enlarged terminal ends in the literature

For a sound comparative frame, we provide interpretive drawings of comparative specimens of extant and fossil specimens that are comparable to our specimens. This is necessary to provide the reader a clear indication which dimensions were measured. Additionally, some of these publications are difficult to obtain. Hence, we also provide a review of available data.

All occurrences of larvae of Scraptiidae with a large terminal end figured in the literature are listed here chronologically; each specimen is numbered consecutively. Cases in which the same specimen has been re-figured are also included chronologically with reference to the original occurrence. While this includes a certain redundancy, it should represent the most comprehensive way of cross-referencing, avoiding interpreting the same specimen as two independent occurrences. While it is always challenging to be sure that such lists are complete, this is an honest attempt to do so. Of course, this is based on earlier attempts such as Young (1987). Larvae of *Anaspis* (Hayashi, Fukuda & Kurosa, 1959; Hayashi, 1962), lacking a prominent terminal end, are not included.

**Figure 4:**
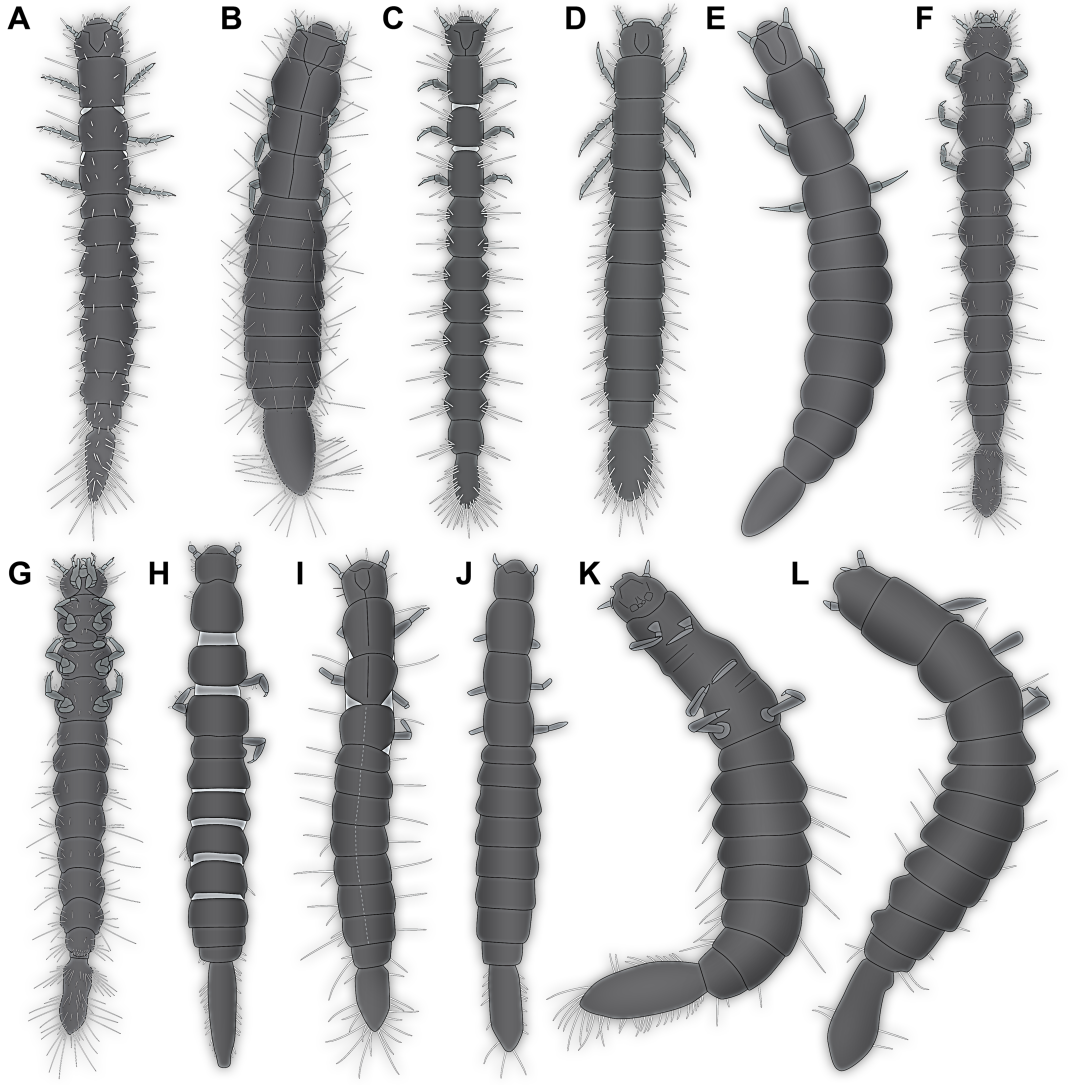
Larvae of Scraptiidae with large terminal ends, redrawn from literature, kindly assisted by Gideon T. Haug, Neuried. (A) Böving & Craighead (1931). (B) Van Emden (1942). (C) Peterson (1951). (D) Hayashi (1980, after Lawrence et al., 1995). (E) Švácha (1995). (F) and (G) Vanin et al. (1996). (H) Lawrence et al. (2011). (I) and (J) bugguide.net. (I) 39846. (J)175620. (K) Weitschat & Wichard (2002). (L) Gröhn (2015).

**Figure 5:**
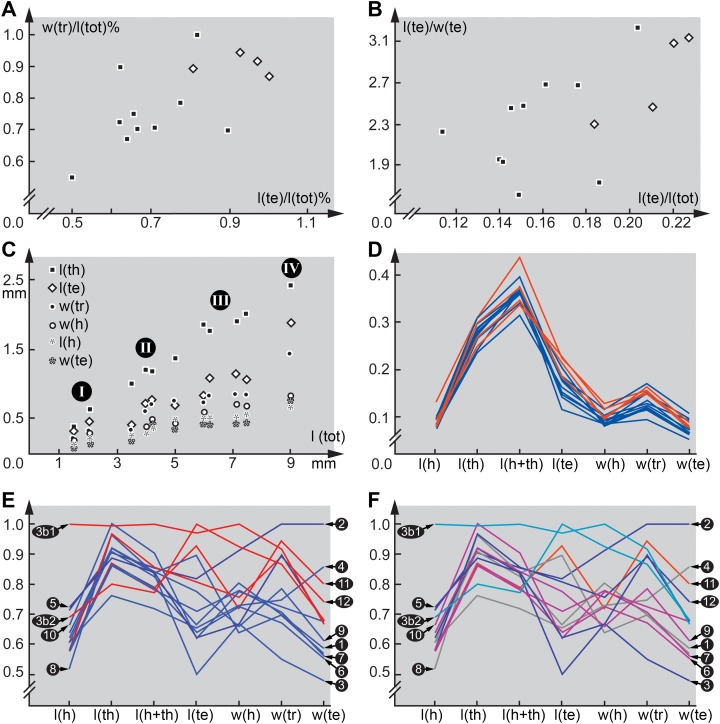
Quantitative aspects of Scraptiidae with large terminal ends. (A) and (B) “Flat” squares are extant larvae; diamonds (squares “on tip”) are larvae from Baltic amber. (A) Ratio of maximum width of trunk w(tr) and total length l(tot) divided by the maximum value of this ratio vs. ratio of trunk end length l(te) and total length l(tot) divided by the maximum value of this ratio. (B) Ratio of trunk end length l(te) and trunk end width w(te) vs. ratio of trunk end length l(te) and total length l(tot). (C) Absolute lengths of various structures (length of thorax l(th), length of trunk end l(te), maximum width of trunk w(tr), width of head w(h), length of head l(h), width of trunk end w(te)) vs. total body length l(tot); note four more or less apparent groups (“proxy instars”). (D) Combinatorial morphospace. Each dimension (named on axis) represents ratio of dimension divided by total body length; blue = extant larvae, red = larvae from Baltic amber. (E) and (F) Combinatorial morphospace. Each dimension (named on axis) represents ratio of dimension divided by total body length and then divided by maximum value. 3b1 and 3b2 represent TripleB larva 1 and TripleB larva 2. (E) Comparison extant–fossil; blue = extant larvae, red = larvae from Baltic amber. (F) Comparison of “proxy instars” based on the results in (C); light blue = I; blue = II; purple = III; red = IV; grey = unclear. Note that the coordinate axes in several panels contain a break to correct for the otherwise long distance to the origin.

1. Böving & Craighead (1931) figured a drawing of a larva of *Scraptia sericea* Melsheimer, 1846 (specimen 1; Fig. 4A) as dorsal habitus view (their plate 44A). Additionally, they provided some details about the mouth parts and the terminal end (their plate 44D). They additionally provide details of the head (dorsal and ventral), mouth parts and the terminal end. The habitus image was re-figured in Hansen (1957). There is no indication of the size of the figured specimen.
2. Van Emden (1942) figured a habitus drawing (specimen 2; Fig. 4B) in dorsal view (his fig. 2) in his determination key under the reference ‘Scraptiidae (*Scraptia*)’. Based on the provided scale the specimen is about 4.2 mm in length.
3. Peterson (1951) figured a drawing of a larva of *Scraptia* sp. (specimen 3; Fig. 4C) as dorsal habitus view (his plate C49G), accompanied by a small detail of the spiracle. The size is partly unclear (due to inconsistencies with the figure caption), yet next to the image it states 3.5 mm. Remark: there are additional editions of this book from 1953, 1957 to 1960. The drawing was re-drawn by Klausnitzer (1978; referencing to the 1957 edition of Peterson) and re-figured (unaltered from Peterson, 1951) in Young (1987).
4. Hansen (1957) re-figured specimen 1 (his fig. 79), i.e. the drawing by Böving & Craighead (1931).
5. Klausnitzer (1978) provided a re-drawing (his fig. H 76) of specimen 3, i.e. the drawing by Peterson (1951). He added shading to the figure and referenced it as ‘Peterson 1957’, hence the third edition of Peterson (1951).
6. Hayashi (1980) apparently figured a larval specimen of *Scraptia* sp. in dorsal view (specimen 4; Fig. 4D). Yet, we were unable to find a copy of this volume (even with help from various colleagues). We only know of the drawing as it was re-figured in Lawrence et al. (1995). We can unfortunately not exclude that Hayashi (1980) figured further specimens.
7. Young (1987) re-figured specimen 3 (his fig. 34.739), the drawing of a larva of *Scraptia* sp. by Peterson (1951). Additionally, he provided a short bibliography of larvae of Scraptiidae. Remark: there is a later edition of Young from 1991.
8. Lawrence et al. (1995) re-figured specimen 4, i.e. the drawing by Hayashi (1980). Please note that there are several different editions of Lawrence et al. which have not all been checked in this study.
9. Švácha (1995) described the larva of *S. fuscula* in detail. A habitus drawing in dorsal view was provided (specimen 5; Fig. 4E). The drawing is not very detailed (his fig. 1), for example omitting setae, but the posture indicates that it is based on an actual specimen. Further details were provided, including a drawing of the head in dorsal view (his fig. 2) and especially numerous SEM images (his figs. 3–22), histological sections documented with light microscopy (his figs. 23–25), TEM images (his figs. 26–29) as well as one scheme showing the proximal region of the terminal end. Yet, only his fig. 1 can be further considered here. One of the SEM images also shows an entire larva (his fig. 3), yet in lateral view, prohibiting measurements of width; also it remains unclear whether it is the same specimen as in his fig. 1. The size of the specimen is given as ‘about five mm’.
10. Vanin et al. (1996) provided a very detailed description of the late larval stages of *S. triangularis*; additionally they provided also information of the pupa and the adult. Besides many details of mouth parts and other structures, they provided habitus drawings of one specimen (specimen 6; Fig. 4F) in dorsal view (their fig. 1) and one specimen (specimen 7; Fig. 4G) in ventral view (their fig. 2). As the lengths of different structures are very different in the two drawings, these are highly likely two different specimens. Specimen 6 measures 7.47 mm, based on the provided scale; specimen 7 measures 7.13 mm. Specimen 6 was re-figured by Lawrence & Ślipiński (2010).
11. Lawrence & Ślipiński (2010) re-figured specimen 6 (their fig. 11.28.3A), i.e. one of the drawings by Vanin et al. (1996).
12. Lawrence et al. (2011) presented a photographic image of a larval specimen of *Scraptia* sp. (specimen 8; Fig. 4H) in dorsal view (their fig. 69C). There is no indication of the size of the figured specimen.
13. Websites are generally not considered to be ‘proper’ scientific sources. Yet given the scarceness of data on larvae of Scraptiidae, we decided to use them here as additional data source. Especially the community ’bugguide’ (https://bugguide.net) is very active and well sorted; furthermore bugguide is hosted by the Department of Entomolgy of the Iowa State University:
  – Image 39846 (© 2005 Jim McClarin) is labelled ‘Bulb-tailed larva’ and nicely shows a photograph of a larva of Scraptiidae in dorsal view (specimen 9; Fig. 4I). Additional images of this specimen are available, but this one was most suitable for measuring. The size of the specimen has been stated to be about ‘6.2 mm maybe’.
  – Image 175620 (© 2008 Jim McClarin) is labelled ‘another scraptiid larva—Canifa’ and shows a photograph of a larva of Scraptiidae in dorsal view (specimen 10; Fig. 4J). Additional images of this specimen are available, but this one was most suitable for measuring. The size of the specimen has been stated to be about six mm.

### Fossil larvae of Scraptiidae with enlarged terminal ends in the literature

Similar to the reports of extant larvae of Scraptiidae, we also summarise the fossil specimens.

14. Weitschat & Wichard (1998) provided a photographic image of a larva of Scraptiidae (specimen 11; Fig. 4K) in Baltic amber (their pl. 63e). No scale or size is provided, but a magnification factor; according to that the specimen is about nine mm in length. Remark: this source was not directly seen by the authors of this publication. According to one of the original authors (W. Wichard, 2019, personal communication) the images are identical to the ones in Weitschat & Wichard (2002; see next point), which the given information is based upon.
15. Weitschat & Wichard (2002) re-figured specimen 11.
16. Gröhn (2015) provided a photographic image of a larva of Scraptiidae (specimen 12; Fig. 4L) in dorsal view (his fig. 7691 on p. 280) preserved in Baltic Amber. It was labelled as ‘Scraptiidae Larve’. The size has been stated to be four mm.

In total, we have ten extant larval specimens of Scraptiidae with an enlarged terminal end available in the literature. Additionally, there are now four fossil specimens known, two from the literature and two from this study. Although not all 400 species of Scraptiidae have such enlarged ends, the number of known larval specimens with large terminal ends is astonishingly low.

### The terminal end

Böving & Craighead (1931) provided a detailed lateral view of the terminal end of the larva. It showed a well separated abdomen segment 9, including a prominent tergite, with a distinctly set off terminal end. Also remains of an abdomen segment 10 were indicated. Notably, the small tergite of the abdomen segment 9 is not apparent in the habitus drawing. Vanin et al. (1996) also mention an abdomen segment 10. Some lines in their drawing in ventral view indicate the presumed abdomen segments 9 and 10; they are shown very small in the drawing. Young (1987) also suggested that the area surrounding the anus should represent abdomen segment 10. Švácha (1995) recognised that the drawings of Böving & Craighead (1931) were in fact erroneously labelled. He provided highly detailed histological sections demonstrating that the supposed tergite of abdomen segment 9 of Böving & Craighead (1931) is that of abdomen segment 8. In the membranous area connecting the abdomen segment with the terminal end, the anal opening is situated.

The anus is ancestrally in Insecta posterior to abdomen segment 11, most likely the last indication of a former telson. As Insecta is an ingroup of Crustacea sensu lato, we should at least expect that similar to modern day eucrustaceans the anus was ancestrally coupled to the telson. In many lineages of Eucrustacea the telson is functionally conjoined to the posterior segment or even several segments, forming a so-called pleotelson, for example in Isopoda. Often this conjoined state of several segments and the telson is caused by non-separation of these structures during ontogeny. Within Insecta it is common to simply consecutively number the abdomen segments. Yet, it is likely that the most posterior unit of the abdomen in many beetle larvae (for example) is in fact a conjoined trunk end not separating the segments and carrying the anus.

Yet, in larvae of Scraptiidae the case is more complicated. In larvae of *Anaspis*, nine discrete units are apparent in the abdomen, the ‘abdomen segment 9’ most likely in fact representing the undifferentiated abdomen segments 9–11 and the remains of the telson. In larvae of *Scraptia* (and other larvae with enlarged terminal ends) the terminal end is often referred to as ‘caudal appendage’ (Švácha, 1995), ‘appendage’ (Van Emden, 1942; Watt, 1987), ‘process’ (Young, 1987; Vanin et al., 1996; Lawrence & Ślipiński, 2010; Lawrence et al., 2011), or interpreted as the abdomen segment 9 (Peterson, 1951; Klausnitzer, 1978).

None of these terms truly reflects the observed morphology. ‘Caudal’ is unfortunate in most metazoans, besides Craniota, as these organisms do not have a cranial-caudal axis due to the lack of a cranium, but an anterior-posterior axis. The term ‘appendage’ is usually very strictly used within Euarthropoda referring to paired, abaxial, ventro-lateral structures developing by a specific gene-regulatory pattern, including structures such as the antennae, mouth parts or legs. The terminal end is unlikely to be a derivative of such a structure. Also ‘process’ is unfortunate as it usually refers to a structure that is not jointed off distinctly, but is continuously drawn out from a surface. It is also unlikely that the terminal end in fact represents the entire abdomen segment 9 (or even more, not differentiated segments), as the anus is not part of this structure.

We cannot contribute new data to this aspect. We can only point out that we apparently do not exactly know to what the prominent end in the larvae corresponds in other forms. We therefore suggest to use a neutral expression, hence ‘terminal end’ to refer to this very prominent structure.

### What is special about the new larvae?

The morphology of the two fossil larvae is unusual. Especially the TripleB larva 1 is very unusual in the shape of the terminal end. In most larvae of Scraptiidae, the shape reminds of a kind of paddle, possibly with the exception of specimen 8 (Lawrence et al., 2011) in which it appears more slender. Yet, in TripleB larva 1 the terminal structure has a distinct constriction further posterior on the terminal end, setting off a posterior region. This region appears almost heart shaped. Outline and symmetry of the posterior region indicate that this is original morphology and not an artefact produced by the process of embedding or diagenesis.

Besides this more qualitative observation there are also important quantitative aspects. First, the two new larvae are smaller than any other so far known larva of Scraptiidae. Second, all modern larvae have a (relatively) shorter terminal end than the two fossils. Also the larva of Scraptiidae figured in Weitschat & Wichard (2002; their fig. 63e) has a rather long terminal end, but slightly shorter than those of the two new fossils. A single modern larva of Scraptiidae reaches an almost comparable ratio of the terminal end versus the remaining body as the larva from Weitschat & Wichard (2002), namely the larva figured in Lawrence et al. (2011, their fig. 69C). Still, this extant larva differs significantly from the fossils and most other modern larvae in having a very slender terminal end, while most other larvae of Scraptiidae have a quite bulbous terminal end.

Furthermore, in some of the scatterplots the fossil larvae plot close together, but somewhat separated from the modern larvae (Figs. 5A and 5B). This could indicate that the Eocene, the time in which Baltic amber originated, still had larval types of Scraptiidae that possessed a morphology that is not represented in the modern fauna. Yet, given the fact that our knowledge on modern larvae of Scraptiidae is still very limited, we can not exclude that we have simply not yet found modern day larvae with a similar morphology. Still, the fact that the few modern larvae show a certain diversity of forms, but all fossil forms plot close together, but outside the space outlined by all modern forms, does not immediately support such an assumption.

### Possible interpretation: the incomplete data on the larval ontogeny of Scraptiidae

It seems well possible that the larvae reported here represent now extinct morphologies. Yet, we also have to consider an alternative interpretation. The two TripleB larvae are significantly smaller than any of the other known forms. Hence, their unusual morphology could be interpreted to be typical for early stage larvae of Scraptiidae that has simply not yet been observed in the modern fauna as these early stages have not yet been found.

Such an interpretation is not supported by plotting the measured dimensions. Scatter plots of absolute values, unfortunately only possible for a smaller sub-set of specimens, provide four more or less distinct ‘clusters’, one with specimens around two mm, a second one around four mm and a third one around seven mm in total length and a single set off specimen with about nine mm (Fig. 5C). While this plot sub-summarises numerous species and a very limited data set, it gives at least an indication that we might face here four instars, or at least a kind of ‘proxy instar’. The increase in size between these is compatible with known size increase within species of Insecta. If the unusual large terminal end (and other aspects) would be only and directly a result of ontogenetic changes, we should expect that all specimens forming one proxy instar plot closer together in the combinatorial morphospace and are at least partly separating from specimens of the others. Also we should expect a cline, i.e. a gradual transition, hence specimens from proxy instar 2 and 3 should plot intermediate between those of 1 and 4. Such a pattern is not observable (Figs. 5D and 5F), therefore it seems unlikely that the special morphology of the new larvae can exclusively be a result of their earlier developmental stage. Most important in this aspect is that specimen 12 (Weitschat & Wichard, 1998) is the largest of the series, but plots close together with the two TripleB larvae. Differences might therefore not be due to an ontogenetic, but to a phylogenetic effect; in the extreme case specimen 12 could be con-specific with TripleB larva 2 and represent an older instar.

### Diversity of larvae of Scraptiidae

The TripleB larva 1 appears also special in other aspects, besides the terminal end (Fig. 5E). Especially the relative length of the head is very different from all other specimens, also width of the head and length of thorax are exceptional. This indicates that the abdomen is rather short in this specimen. While the abdomen is more flexible in larval holometabolans, many beetle larvae, also those of Scraptiidae, have also here sclerotised structures, such as tergites that limit the overall size flexibility in this region, as opposed for example by larvae of Lepidoptera or Diptera. While state of ‘filling’ with food might be a factor here, it cannot easily explain the entire difference. Also it would mean that only this single specimen was starved while all others were well fed. In summary, TripleB larva 1 is quite different from all the other known specimens, not only in having the relatively longest terminal end, but by general differences in overall body ratios and also the shape of the terminal end.

It is furthermore important to note that also not all extant specimens are very similar, but also here we observe quite some variation. As already noted, specimen 8 (Lawrence et al., 2011) is conspicuous by having a rather slender-appearing terminal end. Also, specimen 2 (Van Emden, 1942) is unusual; it appears rather stout, resulting in relative width of trunk and terminal end being the highest ratios measured. This already indicates that there is a still to-be-discovered diversity of form among larvae of Scraptiidae even in the modern fauna. The two TripleB larvae plot outside the morphospace outlined by the modern forms, possibly indicating changes in the morphospace through time; yet, this remains a mere indication.

## Conclusions

It is necessary to increase the sample size significantly to be able to further test the assumption that the diversity of form among larvae of Scraptiidae is larger than what is currently known. Also, the presumed changes in morphospace from the Eocene to today can currently only be assumed. For detecting such changes in larval diversity, further studies of the extant fauna are crucial.

## Supplemental Information

10.7717/peerj.7871/supp-1 Supplemental Information 1 Data set used for quantitative morphological analyses. Measurements of both specimens included in this study plus additional specimens from the literature. Additionally, the presumed (proxy) size group of each specimen is given where possible. Abbreviations (sorted by first occurrence in the table): l(h), length of head; l(th), length of thorax; l(h+th), combined length of head and thorax; l(te), length of terminal end; w(h), maximum width of head; w(tr), maximum width of trunk; w(te), maximum width of terminal end; l(tot), total body length; max, maximum value for each ratio.

## Additional Information and Declarations

### Data Availability

The following information was supplied regarding data availability: The measurements on the larvae as well as ratios calculated from them are available in Table S1. The abbreviations used in the table are explained in the material and methods section of the main text. The specimens used for this study are deposited in the Palaeo-Evo-Devo Research Group Collection of Arthropods, Ludwig-Maximillians-University of Munich, Germany, under repository numbers PED 0006 and PED 0011.
